# CRISPR/Cas9-Mediated Vitellogenin Receptor Knockout Leads to Functional Deficiency in the Reproductive Development of *Plutella xylostella*

**DOI:** 10.3389/fphys.2019.01585

**Published:** 2020-01-23

**Authors:** Lu Peng, Qing Wang, Ming-Min Zou, Yu-Dong Qin, Liette Vasseur, Li-Na Chu, Yi-Long Zhai, Shi-Jie Dong, Li-Li Liu, Wei-Yi He, Guang Yang, Min-Sheng You

**Affiliations:** ^1^State Key Laboratory of Ecological Pest Control for Fujian and Taiwan Crops, Fujian Agriculture and Forestry University, Fuzhou, China; ^2^Institute of Applied Ecology, Fujian Agriculture and Forestry University, Fuzhou, China; ^3^Key Laboratory of Integrated Pest Management for Fujian-Taiwan Crops, Ministry of Agriculture, Fuzhou, China; ^4^Fujian Provincial Key Laboratory of Insect Ecology, Fujian Agriculture and Forestry University, Fuzhou, China; ^5^Department of Biological Sciences, Brock University, St. Catharines, ON, Canada

**Keywords:** diamondback moth, mutant lines, CRISPR/Cas9, Vg transport, embryonic development, reproductive regulation

## Abstract

The vitellogenin receptor (VgR) belongs to the low-density lipoprotein receptor (LDLR) gene superfamily and plays an indispensable role in Vg transport, yolk deposition, and oocyte development. For this reason, it has become a promising target for pest control. The involvement of *VgR* in Vg transport and reproductive functions remains unclear in diamondback moths, *Plutella xylostella* (L.), a destructive pest of cruciferous crops. Here, we cloned and identified the complete cDNA sequence of *P. xylostella VgR*, which encoded 1805 amino acid residues and contained four conserved domains of LDLR superfamily. *PxVgR* was mainly expressed in female adults, more specifically in the ovary. *Px*VgR protein also showed the similar expression profile with the *PxVgR* transcript. CRISPR/Cas9-mediated *PxVgR* knockout created a homozygous mutant of *P. xylostella* with 5-bp-nucleotide deletion in the *PxVgR*. The expression deficiency of *Px*VgR protein was detected in the ovaries and eggs of mutant individuals. Vg protein was still detected in the eggs of the mutant individuals, but with a decreased expression level. However, *PxVg* transcripts were not significantly affected by the *PxVgR* knockout. Knockout of *PxVgR* resulted in shorter ovarioles of newly emerged females. No significant difference was detected between wild and mutant individuals in terms of the number of eggs laid in the first 3 days after mating. The loss of *PxVgR* gene resulted in smaller and whiter eggs and lower egg hatching rate. This study represents the first report on the functions of *VgR* in Vg transport, ovary development, oviposition, and embryonic development of *P. xylostella* using CRISPR/Cas9 technology. This study lays the foundation for understanding molecular mechanisms of *P. xylostella* reproduction, and for making use of *VgR* as a potential genetic-based molecular target for better control of the *P. xylostella.*

## Introduction

In insect reproduction, vitellogenesis is a central event that involves the production and secretion of the yolk protein precursor vitellogenin (Vg) in the fat body (Tufail and Takeda, 2009). This is followed by Vg internalization of developing oocytes from the hemolymph through the endocytosis of the vitellogenin receptor (VgR), and dissociation of VgR which is returned to the oocyte surface, while Vg is transported to the oocytes (Raikhel and Dhadialla, 1992; Mukherjee et al., 1997). VgR is the specific receptor in Vg uptake and dominates the oocyte maturation and reproductive process of insect species (Goldstein et al., 1979; Sappington et al., 1995). Functional deficiency of VgR through genetic mutations or RNA interference can produce phenotypes resulting in yolkless (Schonbaum et al., 1995; Ciudad et al., 2006) or “restricted-ovulator” (Schjeide et al., 1976). Understanding the possible use of such mutants may help develop strategies for pest control.

In insects, VgRs are considered to be members of the low-density lipoprotein receptor (LDLR) superfamily, which encodes for a large protein of 180–214 kDa (Sappington and Raikhel, 1998). Many proteins of the LDLR are characterized by five amino acid domains: (1) ligand-binding domains (LBDs) with cysteine-rich class A (LDLR_A_) repeats, (2) the epidermal growth factor (EGFs)-like domain (EGFD), which contains cysteine-rich class B (LDLR_B_) repeats, (3) an O-linked sugar domain (OLSD) containing rich serine/threonine, (4) a transmembrane domain (TMD) involved in the receptor anchoring, and (5) a cytoplasmic domain (CPD) for the internalization signal in receptor localization (Goldstein et al., 1985). Despite their common structural elements, there are some differences in their molecular functions (Herz and Bock, 2002; Strickland et al., 2002).

As a key transporter of insect Vgs, VgR may be a promising target for pest control (Sappington and Raikhel, 1998; Shu et al., 2009). An increasing number of studies has explored the *VgR* functions in insects using RNA interference technology (RNAi). For example, *VgR* knockdown through RNAi can significantly suppress egg production and ovary development (Lu et al., 2015; Moriyama et al., 2016; Zhang et al., 2016; Han et al., 2017). However, an effective RNAi is difficult to obtain in some insects, especially in Lepidoptera (Terenius et al., 2011). RNAi may not always work to identify the binding specificity of VgR with Vg. CRISPR/Cas9, originally originated from an adaptive defense formed by bacteria and archaea, is increasingly used as a cutting-edge tool for gene editing in eukaryotes (Cong et al., 2013; Knott and Doudna, 2018). This technique has shown high potential in gene functions analysis in insects, such as Lepidoptera (Zhang et al., 2015; Huang et al., 2016; Wang et al., 2016; Guo et al., 2019), and in the development of pest control systems (Hammond et al., 2016; Reid and O’Brochta, 2016). Despite the need to further understand the roles of *VgR* gene in Vg transportation and insect reproduction, studies to verify the functions of *VgR* gene using CRISPR/Cas9 are lacking.

The diamondback moth, *Plutella xylostella* (L.) (Lepidoptera, Plutellidae), is a proliferous, worldwide pest, mainly attacking cruciferous crops (Furlong et al., 2013). It has become a study model for insect pests and much interest has led to a better understanding of its development and molecular biology to reduce its reproductive capacity. In this study, the molecular characteristics of *P. xylostella VgR* (*PxVgR*) were identified, and its spatial and temporal expression were analyzed. CRISPR/Cas9-mediated knockout of *VgR* was then conducted to investigate its functions in Vg transportation and reproductive development of *P. xylostella*. This is a first report on the functions of *VgR* on reproductive regulation in *P. xylostella*, even in insect species using CRISPR/Cas9 technology, which could be used to investigate potential genes or regulatory mechanisms on which techniques can be developed to control pest populations.

## Materials and Methods

### Insect Culture

The insecticide susceptible *P. xylostella* colony Geneva 88 (thereafter called G88) used for this study was obtained from Cornell University in 2016 and has since been reared in the greenhouse at the Fujian Agriculture and Forestry University. Larvae were reared on the fresh artificial diet (#F9772-DBM, Frontier Scientific Services, United States) at 25 ± 1°C, 65 ± 5% RH and L: D = 16:8 h and pupae were transferred into a box (10.4 cm × 7.3 cm × 8.5 cm) until eclosion. After emergence, adults were fed with 10% honey solution for nutrition.

### Total RNA Isolation and cDNA Synthesis

Total RNA was isolated from individuals or tissues with the TRIzol Reagent (Invitrogen, United States). The purity of RNA samples was verified using the Nano Vue spectrophotometer (GE-Healthcare) and detected with agarose gel electrophoresis. Then, the cDNA was synthesized by Hiscript^TM^ Reverse Transcriptase (Vazyme, China) with an amount of 500 ng RNA.

### *PxVgR* Cloning

The candidate sequence obtained from the *P. xylostella* Genome Database^1^ was authenticated by segmented PCR using specific primers designed with Primer tool at https://www.ncbi.nlm.nih.gov/tools/primer-blast/ (Table 1). PCR reaction was executed using the following procedure: 95°C, 3 min, and then executing 34 cycles, including 95°C, 30 s, 55–58°C, 30 s, and 72°C, 2 min, and finally with an extension at 72°C for 5 min. The PCR of 3′-RACE was also implemented using FirstChoice^®^ RLM-RACE Kit (Invitrogen, United States) according to the protocol of the manufacturer for the amplification on 3′ sequence. All PCR products were purified and linked with the pJET1.2 vector (Thermo, United States) for sequencing.

**TABLE 1:** Primers used for this study.

Primer name	Primer sequence 5′-3′
*VgR* F1	TCTTTTCTGCACACTTTTAGGG
*VgR* R1	GGGTCTCGTTGTACTCGTCG
*VgR* F2	CTACTGCTGCTCTGTCTAGCG
*VgR* R2	GGGCTGGTCTCGTGGATAAG
*VgR* F3	CAGGGTTCTACTGACGCTGG
*VgR* R3	AGGGCAGTCATCTATCCCGT
*VgR* F4	TACTACATGGGCTACACCTGC
*VgR* R4	GAGCAGAGGTACTCGCAGTC
*VgR* F5	CGACCATCAATCCACCCCAA
*VgR* R5	CCCAGTCTACTGCCACCTTG
3′Race	ACAGACATAGACGAGTGCCG
*RIBP* F	CAATCAGGCCAATTTACCGC
*RIBP* R	CTGCGTTTACGCCAGTTACG
qRT-PCR F	ATTGTGACCCCGATGGACTG
qRT-PCR R	TGCAGCGGGTCTCATTCATAG
sgRNA-F1^∗^	TAATACGACTCACTATAGGAGGCTCCGTGGCGGCGTGCG **GTTTTAGAGCTAGAAATAGCAAGTTAAAATAAGGCTAGTCC**
sgRNA-sgR	AAAAGCACCGACTCGGTGCCACTTTTTCAAGTTGATAACGGA CTAGCCTTATTTTAACTTGCTATTTCTAGCTCTAAAAC
*VgR* sgF1	TGGACTGGGTTCTGACCCTAT
*VgR* sgR1	TTGTAACCCTCCTTGCCAGA

*^∗^T7 promoter sequence is underlined, and sgRNA skeleton sequence is in bold.*

### Sequence Comparison and Phylogenetic Analysis of *Px*VgR

The fragments of *PxVgR* were assembled using DNAMAN 8.0 software. ORF finder^2^ was used to predict the open reading frame. The amino acid sequence of *PxVgR* was inferred using the translation tool on the Snapgene 2.3.2. SignalIP 4.1 Server^3^ was used to predict the signal peptide. The molecular mass and isoelectric point were determined through the ExPASy proteomics server^4^. The InterPro^5^ was used to analyze functional domains and conserved domains. The transmembrane regions were predicted by TMHMM Server v.2.0^6^, and the GPP Prediction Server^7^ was used for the analysis of the O-linked glycosylation sites. The homologous VgRs sequences were aligned by Clustal X 2.0, and the phylogenetic tree was constructed with the neighbor-joining method using the MEGA 5.1.

### Expression Analysis of *Px*VgR

For stage- and sex-specific expression profiles, all stages [eggs, larvae (from L1 to L4), pupae, and adults] and both sexes (from fourth instar to adult) were sampled [three samples of 50–100 mg (i.e., pooled individuals) per stage and sex]. For tissue-specific expression patterns, 100 newly emerged adults were dissected to extract head, thorax, fat body, midgut + malpighian tubule, ovary, and epidermis tissues in DNase and RNase free water (QIAGEN, Germany) (Peng et al., 2019). Each tissue of these individuals was then separately put in RNA Stabilization Reagent (RNAlaterTM, Qiagen, Germany) and stored at −80°C. RNA isolation and cDNA synthesis of each sample was completed as described above. qRT-PCR was performed with GoTaq^®^ qPCR Master Mix Kit (Promega, United States). Each reaction consisted of 2 μL cDNA template (100 ng/μL), 10 μL 2 × real-time PCR Mix (containing SYBR Green I), 0.4 μL of each primer (10 mmol/L), and 7.2 μL nuclease-free water. The qPCR program included 95°C, 30 s; then 95°C, 5 s, and 60°C, 30 s for 44 cycles. The homogeneity of PCR products was detected by melting curve analysis. Ribosomal protein L32 gene (*RIBP*) as the internal reference was used to normalized the gene expression levels according to the comparative Ct method (2^–ΔCt^), which is the best method for calculating the mean ± SE. for each group as individual data points (Schmittgen and Livak, 2008; Peng et al., 2017). Specific primers used for the qPCR were listed in Table 1.

### Protein Preparation and Western Blot

Partial sequences from 309 to 1188 bp of *PxVgR* and 4251 to 5271 bp of *PxVg* were PCR amplified and cloned into pJET1.2 vector. The recombinant plasmid of PJET1.2-VgR/Vg was transferred into *E. coli* DH5α and cultured for 12 h in LB culture medium. The plasmid was then extracted and sent to ABclonal Biotech company (Wuhan, China) for primary polyclonal antibody (anti-VgR/anti-Vg) synthesis.

Proteins from the various developmental stages and tissues were extracted according to Wang et al. (2003). Each protein sample was dissolved in a buffer (8M Urea in Tris–HCl with a PH of 8.0, 1% SDS) for 60∼90 min at 50°C, and protein contents were detected using BCA Protein Quantification Kit (Strong) (Yeasen, China). For Western blot, the same amounts of the total proteins of each sample (25 ug) were separated with 10% SDS-PAGE, and then transferred to polyvinylidene difluoride (PVDF) membrane (Millipore, United States) for 1.5 h under 350 mA. The membrane was then blocked with 3% (w/v) BSA in 1 × TBST overnight at 4°C. It was followed by an incubation with Tubulin antibody (1:1000) (Sigma-Aldrich, United States) and *Px*VgR primary antibody (1:1000) or *Px*Vg primary antibody (1:1000) at room temperature for 2 h, before being washed three times with 1 × TBST for 10 min each time. Finally, the membrane was incubated again with the secondary antibody (1:5000) of HRP-labeled goat anti-rabbit IgG (H + L) (Affinity Biosciences, OH) at room temperature for 45 min and washed as described above. Chromogenic reactions were performed with Clarity Western ECL Substrate kit (Bio-Rad, China) and photographed using Fusion Fx Vilber Lourmat (Fusion Fx5, Vilber, French).

### sgRNA Design and *in vitro* Transcription of sgRNA and Cas9

**FIGURE 1:**
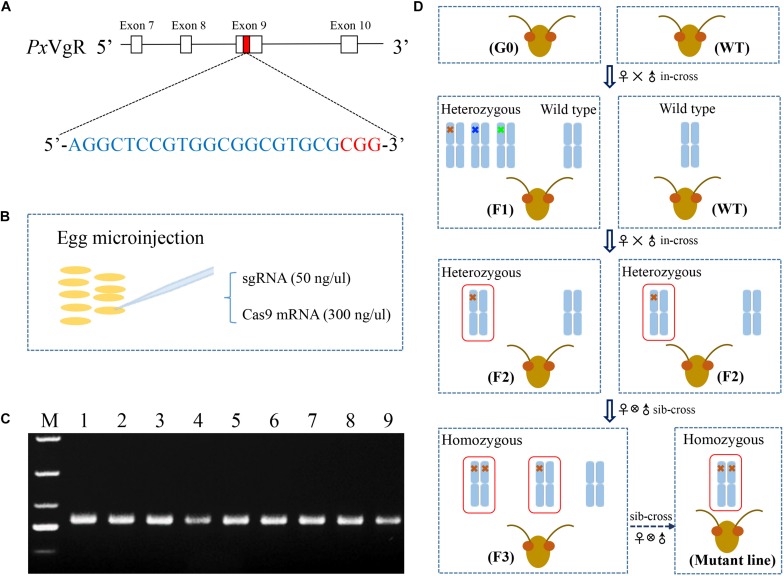
The strategy used for the knockout of *VgR* gene in *P. xylostella* using the CRISPR/Cas9 system. **(A)** The sgRNA targeting site was located on the exon 9 of *PxVgR* gene. The sgRNA-targeting sequence was highlighted in blue, and the protospacer-adjacent motif (PAM) sequence in red. **(B)** A mixture of sgRNA and Cas9 was injected into the fresh preblastoderm-stage eggs using microinjection system. **(C)** The gDNA fragment of *PxVgR* (432 bp) was amplified to detect the mutations in the target region (Lanes 1–9). **(D)** The flow chart of the crossing program to construct the homozygous *PxVgR* mutant line. The mutations were detected with Sanger sequencing of parental individuals of *P. xylostella*, and the mutant genotypes were further identified using TA cloning and sequencing. Blue columns represent the autosomes, which indicated by one or two crosses were heterozygous or homozygous mutations, respectively. The crosses in different colors mean different genotypes, and 5-bp deletion was showed with red cross. The red boxes denoted the autosomal regions with an edited *PxVgR* gene.

Based on the principle of N20NGG (Jinek et al., 2012), the exon 9 of *PxVgR* was selected to design the target site (5′-AGGCTCCGTGGCGGCGTGCG-3′) via the CRISPR gRNA Design tool-ATUM^8^ (Figure 1A). The 5′ end of the target sequence in sgRNA was added to the bases of GG to ensure *in vitro* transcription stability by the T7 RNA polymerase (Li et al., 2017). Two oligonucleotides were used as primers in the PCR reaction using PrimeSTAR^®^ HS DNA Polymerase (TaKaRa, China) (Bassett et al., 2013). A specific oligonucleotide encoding the binding site of T7 polymerase and the sgRNA target sequence N20 was used as the forward primer, and a universal oligonucleotide encoding the remaining sgRNA was used as the reverse primer (Table 1). PCR products were purified with Universal DNA Purification Kit (TIANGEN, China), and their concentration and purity were detected with NanoVue spectrophotometer (GE-Healthcare) and agarose gel electrophoresis, respectively. Subsequently, the sgRNA was *in vitro* synthesized with HiScribe T7 Quick High Yield RNA Synthesis Kit (New England Biolabs, United States) following the manufacturer’s protocol. The linearized pTD1-T7-Cas9 was used to synthesize the Cas9 mRNA with HiScribe T7 ARCA mRNA Kit (with tailing) (New England Biolabs, United States) (Huang et al., 2016). sgRNA (*VgR*) and Cas9 were further purified, respectively, with RNA Extraction Reagent (Solarbio, China).

### Egg Collection and Microinjection

During the oviposition of females, fresh eggs at preblastoderm stage were collected with 10 cm^2^ parafilm sheets precoated with the extract of cabbage leaf, and the sheets were changed for new ones every 30 min, according to Huang et al. (2016). Mixtures of sgRNA (50 ng/μl) and Cas9 (300 ng/μl) were injected into the posterior pole of fresh preblastoderm-stage eggs (Figure 1B) within 1 h of oviposition, using Olympus SZX16 microinjection system (Olympus, Japan). The injected eggs were maintained at 25 ± 1°C, 60 ± 10% RH in a dark environment for hatching.

### Genomic DNA Isolation and PCR-Based Genotyping

Genomic DNA (gDNA) was isolated from individual adults, using TIANamp Genomic DNA Kit (TIANGEN, China), following the manufacturer’s instructions. The gDNA fragments of *PxVgR* (432 bp) (Figure 1C) were amplified with the specific primer pairs showed in Table 1. PCR reaction with the total volume of 25 μL contained template gDNA (1 μL), PrimeSTAR Max premix (2×) (13.5 μL, including 12.5 μL Max buffer, 0.5 μL dNTP Mix, and 0.5 μL DNase), each primer (1 μL), and ddH_2_O (8.5 μL). The PCR protocol was as follows: one cycle of 95°C for 3 min, and 95°C for 30 s, 55°C for 30 s, 34 cycles of 72°C for 30 s, and one cycle of 72°C for 5 min. Subsequently, the PCR products were sequenced to detect the mutations, and the products with mutations were linked with the pJET1.2/blun vector (Thermo, China) to confirm the mutant types of *PxVgR* (insertion or deletion).

### The Construction of Homozygous *PxVgR*-Mutant Strain

A serial crossing scheme was designed to establish a stable homozygous mutant strain of *VgR* gene (Figure 1D). The preblastoderm-stage eggs microinjected with sgRNA and Cas9 mixture were developed to adulthood as the initial generation 0 (G0). The virgin G0 adults were single-mated with wild types (WT) to produce a transgenic line (F1), and genotyped by sequencing. F1 individuals produced by the mutant G0 adults were reared to eclosion and in-crossed with WT to obtain F2 offspring. F1 adults were also single-sequenced to verify the mutations, and F2 individuals produced by heterozygous F1 with the same allelic mutation were collected and sibling-crossed to produce F3 progeny, those of which with the homozygous mutations were preserved to develop a stable homozygous mutant strain of *VgR* (MUT).

### Immunofluorescence Assay

The newly emerged females from MUT and WT strains were anesthetized with CO_2_, and dissected in phosphate buffered saline (PBS) to obtain ovaries. Dissected ovaries were fixed for 30 min with 4% (vol/vol) paraformaldehyde. After washing with 1 × TBST five times, samples were soaked in 0.1% (vol/vol) Triton X-100 for 30 min at room temperature. Next, all samples were kept in 3% (w/v) BSA in 1 × TBST for 60 min, as well as incubated with the *Px*VgR antibody (1:200) at room temperature for 2 h. They were then washed with 1 × TBST 10 times. For immunofluorescence, the samples were incubated using Alex Fluor Plus 594-conjugated secondary antibody (goat anti-rabbit, Invitrogen) (1:200) at 4°C overnight and rinsed with 1 × TBST five times, then dyed with DAPI Fluoromout-G^TM^ staining (contain DAPI, Em = 455 nm, Yeasen). Finally, fluorescence reaction was examined with the Leica SP8 confocal microscope (Leica, Germany).

### Proteomic Identification of *P. xylostella* Eggs

To further test whether Vg could still enter into the eggs after *VgR* knockout, the egg protein of *P. xylostella* from the *VgR* mutant strain was prepared as previously described in Wang et al. (2003), and a 25 μg sample was separated with 10% SDS-PAGE. After being stained with Coomassie blue, the gel section containing the proteins with molecular weights of 120∼245 kDa were excised and sent to BGI-Shenzhen (Shenzhen, China) for proteomic identification. The gel section was digested with Trypsin Gold (Promega, United States) and analyzed using high-performance liquid chromatography and mass spectrometry (HPLC-MS/MS) according to the BGI’s protocol. The acquired data were converted by Proteome Discoverer 1.3, and then searched with Mascot v2.3.02 against the NCBI_ *Plutella xylostella* database (21065 sequences) to identify the proteins.

### Ovary Development and Fecundity Analysis of *P. xylostella*

Fifteen ovaries were dissected from newly emerged MUT and WT females as previously described and washed with PBS three times. Their ovary morphological characteristics were photographed by digital microscope VHX-2000C (KEYENCE, Japan), and the length of each ovariole was measured.

Finally, the newly emerged MUT and WT female adults were collected, and each female was single-mated with a newly emerged WT male in a plastic cup. A hole was drilled in the lid to introduce the parafilm sheets, with the cabbage leaf extract and 10% honey solution was provided for nutrition. The number of eggs was recorded within 3 days of mating; the size of eggs was measured and hatchability was calculated. The fecundity of twenty-five pairs of *P. xylostella* from the two strains were analyzed.

### Data Analysis

Data of the hatching rate were arcsine-transformed to meet assumption of normality. Multiple comparisons were carried out using one-way analysis of variance (ANOVA) with Tukey HSD multiple test. The comparison between MUT and WT strains was performed using an independent sample *t*-test. Data analyses were completed by SPSS v. 19.0 software (SPSS Inc., United States).

## Results

### Identification and Analysis of *PxVgR*

**FIGURE 2:**
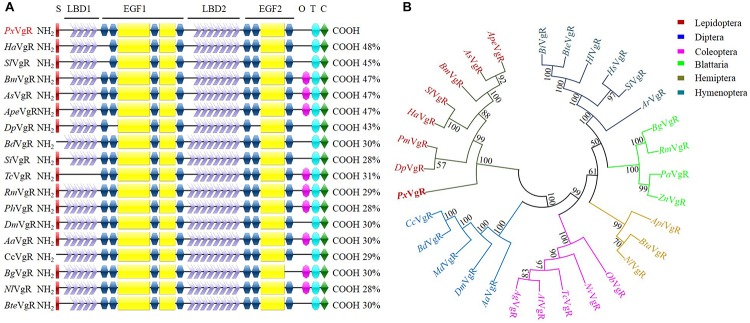
Sequence comparison and phylogenetic tree of insect VgRs. **(A)** Diagrammatic comparison of typical domains between *Px*VgR and other insects VgRs. The percentages (%) at the right of each sequence indicated the identify compared to *Px*VgR. **(B)** Phylogenetic tree of insects VgRs based on the method of neighbor-joining (NJ) with a bootstrap value of 1000 replicates. Sequences were deposited in the GenBank database, which included the VgRs of *Plutella xyllostella* (*Px*VgR, MN044389), *Actias selene* (*As*VgR, AFV32171), *Acyrthosiphon pisum* (*Api*VgR, XP_016657813), *Aedes aegypti* (*Aa*VgR, AAK15810), *Aethina tumida* (*At*VgR, XP_019881581), *Anoplophora glabripennis* (*Ag*VgR, XP_018579962), *Athalia rosae* (*Ar*VgR, XP_012266547), *Antheraea pernyi* (*Ape*VgR, AEJ88360), *Bactrocera dorsalis* (*Bd*VgR, AGE83235), *Bemisia tabaci* (*Bta*VgR, ADM34986), *Blattella germanica* (*Bg*VgR, CAJ19121), *Bombus impatiens* (*Bi*VgR, XP_012241122), *Bombus terrestris* (*Bte*VgR, XP_003402703), *Bombyx mori* (*Bm*VgR, ADK94452), *Ceratitis capitata* (*Cc*VgR, JAC05586), *Danaus plexippus* (*Dp*VgR, OWR52293.1), *Drosophila melanogaster* (*Dm*VgR, AAB60217), *Habropoda laboriosa* (*Hl*VgR, KOC62359), *Harpegnathos saltator* (*Hs*VgR, XP_011139074.1), *Helicoverpa armigera* (*Ha*VgR, AGF33811), *Musca domestica* (*Md*VgR, XP_19894781), *Nicrophorus vespilloides* (*Nv*VgR, XP_017771582), *Nilaparvata lugens* (*Nl*VgR, ADE34166.1), *Oryctes borbonicus* (*Ob*VgR, KRT81424), *Papilio machaon* (*Pm*VgR, KPJ08910), *Pediculus humanus corporis* (*Ph*VgR, XP_002423121.1), *Periplaneta americana* (*Pa*VgR, BAC02725), *Rhyparobia maderae* (*Rm*VgR, BAE93218), *Solenopsis invicta* VgR (*Si*VgR, AAP92450), *Spodoptera litura* (*Sl*VgR, ADK94033), *Tribolium castaneum* (*Tc*VgR, XP_15837722), *Zootermopsis nevadensis* (*Zn*VgR, XP_021934248). S, signal peptide; O, O-linked sugar domain; T, transmembrane domain; C, cytoplasmic domain.

The *VgR* sequence of *P. xylostella* contained an ORF of 5418 base pairs (bp) encoding 1,805 amino acids (*PxVgR*, GenBank accession no. MN044389). The deduced protein had a signal peptide (MHKKQLIAGLLLLCLAATAA) located in N-terminal (Figure 2A and Supplementary Figure S1). The theoretical molecular weight was estimated at 199.9 kDa with an isoelectric point of 5.49. Four conserved domains belonging to the LDLR superfamily receptors were detected in *Px*VgR: two ligand-binding domains (LBDs), which included four class A (LDLR_A_) repeats in the first (LBD1) and seven in the second (LBD2) domains; two types of epidermal growth factor precursor homology domains (EGF 1/2), closely followed by LBD1/2; a hydrophobic single-pass transmembrane domain (TMD) (GAWVPLLVGTFVLMAVCFGIVVL) located at amino acid positions 1682–1704 aa, and; the cytoplasmic domain (CPD) of 1705–1805 aa in C-terminus (Figure 2A and Supplementary Figure S1). However, there was no O-linked sugar domain (O) predicted in *Px*VgR.

### Structural Comparison, Sequence Alignment, and Phylogenetic Analysis of *Px*VgR

*Px*VgR had a high degree of the structural similarity with other insect VgRs. The differences were only detected in LDLR repeat numbers of LBD motifs (Figure 2A). There were four A repeats classes in LBD1 and seven in LBD2 of *Px*VgR, while five and eight repeats were included in LBD1 and LBD2 of some other insect VgRs (Figure 2A). BLAST-X searches (NCBI) showed that *Px*VgR had the most similarity with *Helicoverpa armigera* VgR (48%), following by *Bombyx mori, Actias selene*, and *Antheraea pernyi* (47%) (Figure 2A and Supplementary Figure S1). According to the phylogenetic analysis, all families were highly conserved, including *Px*VgR that was classified into the same cluster with other Lepidoptera species (Figure 2B).

### Expression Profile of *Px*VgR

**FIGURE 3:**
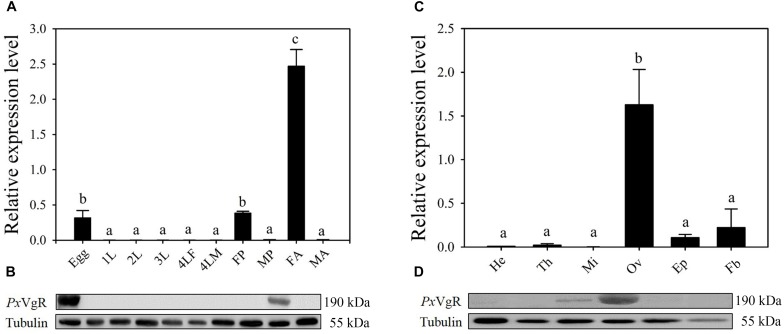
The developmental and tissue-specific expression patterns of the *Px*VgR in *P. xylostella.* **(A,B)** The developmental expression profiles of *PxVgR* transcripts and protein were analyzed by qRT-PCR and Western blot, respectively. The mRNA level was normalized to the *P. xylostella RIBP* transcripts in qRT-PCR analysis; Proteins (25 ug/lane) were separated using SDS-PAGE, then blotted to the PVDF membrane and probed with the polyclonal antibody against *Px*VgR. Tubulin was used as the internal reference. Egg; 1-3L: 1-3 instar larvae; 4LM/4LF: 4th instar male/female larvae; MP/FP: male/female pupae; MA/FA: male/female adults. **(C,D)** The tissue-specific expression patterns of *PxVgR* transcripts and protein were also analyzed by qRT-PCR and Western blot, respectively. He, head; Th, thorax; Mi, midgut + malpighian tubule; Ov, ovary; Ep, epidermis; Fb, fat body. Data represent with three biological replicates and each replication repeat three times. The bars were shown as the mean ± SE. Different letters mean significant differences (*P* < 0.05).

The developmental expression profiles showed that *PxVgR* had the highest expression in female adults, low levels in eggs and female pupae, and negligible levels in all other stages (Figure 3A). Western blot analysis also revealed that the *Px*VgR protein was detected in eggs and female adults (Figure 3B). Tissue-specific expression profiles showed that *PxVgR* gene was specifically expressed in ovaries when compared with the head, thorax, midgut + malpighian tubule, epidermis, and fat body (Figure 3C). *Px*VgR protein also showed a similar expression profile with the *PxVgR* transcript detection (Figure 3D).

### The Mutation of *PxVgR* Produced by the CRISPR/Cas9 System

**FIGURE 4:**
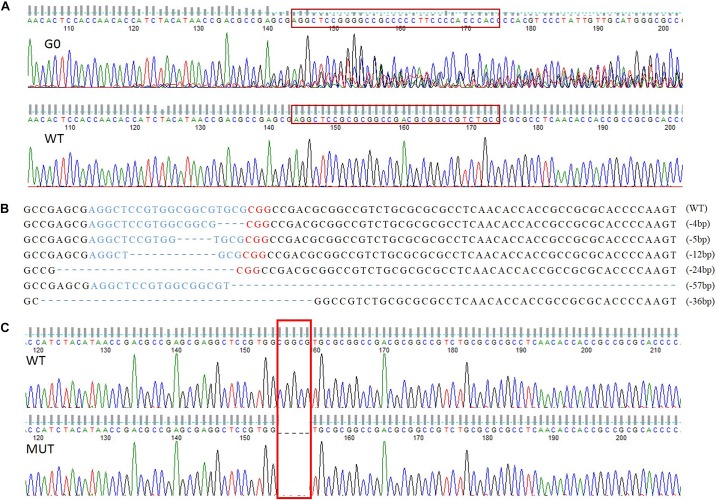
The sequence-specific mutant genotypes of *PxVgR* identified in F1 generation of *P. xylostella* based on CRISPR/Cas9. **(A)** Targeted sequences were highlighted in the red box. **(B)** The sgRNA-targeting sequence was represented by blue letters, and the PAM sequence was in red. The deleted bases were displayed as dashes, and the numbers of deleted bases were demonstrated at the right of each allele (–, deletion). **(C)** 5 bp deletion were highlight with red box, which was further used to construct homozygous line.

A total of 258 preblastoderm eggs of *P. xylostella* were microinjected with sgRNA and Cas9 mixture, with 58.9% (152/258) of these eggs hatching, and 80.3% (122/152) of these larvae successfully developing into the adult stage (G0). Subsequently, sequencing analysis of 122 G0 individuals showed a high mutation efficiency (63%) in the target site of the *PxVgR* gene through CRISPR/Cas9. This result was verified using the sequencing chromatogram with the obvious multi-peaks at target sites (Figure 4A).

### Establishment of the Homozygous *PxVgR* Mutant Line

Six different deletion types were identified in F1, including four individuals with 4 bp, one with 5 bp, two with 12 bp, three with 24 bp, two with 36 bp, and one with 57 bp (Figure 4B). Subsequently, the offspring produced by the parents (F1) with 4 and 5 bp deletion were retained as F2, and developed the sib-cross strain. The sib-cross pairs with single homozygosity (aa♀ Aa♂/aa♂ Aa♀) were screened in F3, of which offspring were continuously sib-crossed to produce the stable homozygous mutant line of *PxVgR*. The sequencing results of F4 pairs showed that approximately 18.2% of them (6/33 pairs) were double homozygotes (aa♀ aa♂) for 5 bp deletion at the target site of *PxVgR* (Figure 4C).

**FIGURE 5:**
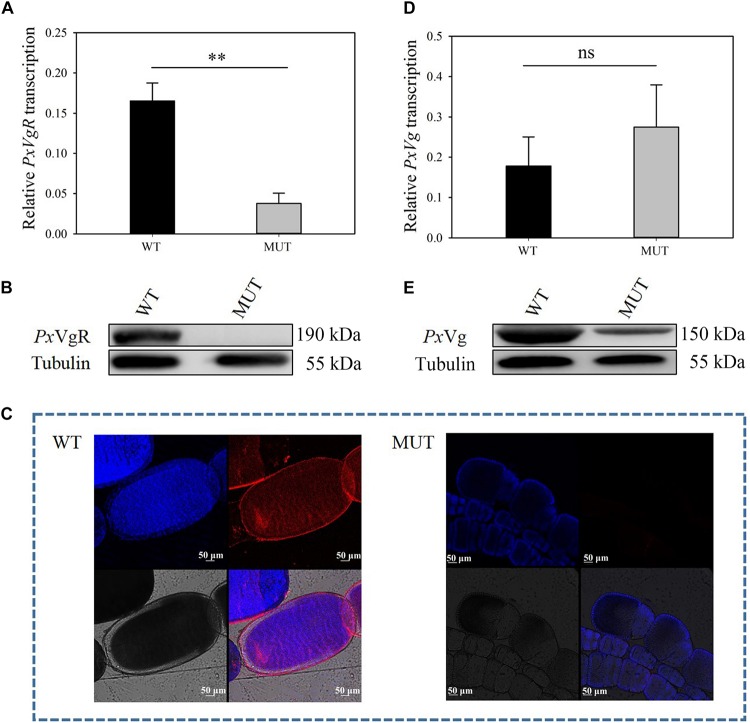
The effects of *VgR* knockout on the expression of *Px*VgR and *Px*Vg in *P. xylostella*. **(A,D)** The transcription level of *PxVgR* and *PxVg* genes were analyzed by qRT-PCR, however, **(B,E)** the expression patterns of *Px*VgR and *Px*Vg proteins were analyzed by Western blot. **(C)** The ovaries dissected from newly emerged MUT and WT females were successively treated with the *Px*VgR polyclonal antibody and Alex Fluor Plus 594-conjugated-secondary antibody (goat anti-rabbit) (red), and stained with DAPI Fluoromout-G^TM^ for DNA (blue); bar = 50 um. The results were shown as mean ± SE. The asterisk ^∗∗^ above the bars represented significant difference (*P* < 0.01).

The mutation efficiency was evaluated based on mRNA expression, protein content, immunoreactivity, and proteomic identification. The transcripts of *PxVgR* were effectively suppressed after knockout of *VgR* (*t* = 4.942, df = 4, *P* = 0.008) (Figure 5A). Furthermore, no protein was detected in the mutant individuals using Western blot analysis, as well as in the ovaries using immunofluorescence (Figures 5B,C). According to the analysis of egg proteins of ∼120–245 kDa separated by SDS-PAGE using mass spectrometry, we also found that VgR was still not detected in the eggs of MUT strain *P. xylostella* (Supplementary Figure S2).

### The Effects of *VgR* Knockout on Vg Uptake of *P. xylostella*

To investigate the functions of VgR on Vg transportation in *P. xylostella*, the Vg expression and deposition was detected after knocking out of *PxVgR*. qRT-PCR analyses showed that there was no significant effect on *PxVg* transcription (*t* = 0.758, df = 4, *P* = 0.419) (Figure 5D). Vg protein was still detected in the eggs of *P. xylostella* MUT strain, but with a decreased expression level compared to the WT strain (Figure 5E). Mass spectrometry further confirmed that there were also some tryptic peptides specific to *Px*Vg protein in the *VgR* MUT strain (Supplementary Figures S2, S3), indicating that some Vg could still be transferred into eggs after *VgR* deletion.

### The Effects of *VgR* Knockout on *P. xylostella* Reproduction

**FIGURE 6:**
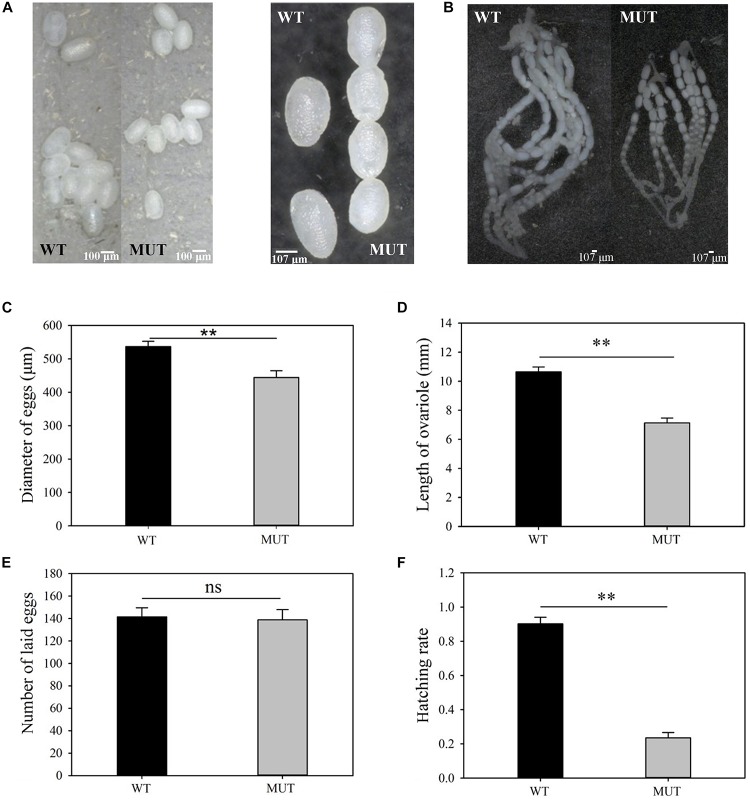
The effects of *VgR* knockout on ovary development and reproduction in *P. xylostella*. **(A)** The eggs were collected within 3 days of mating, and **(B)** the ovaries were dissected from newly emerged MUT and WT females; (**A**, left) bar = 100 um; (**A**, right), **(B)** bar = 107 um, **(C)** Diameter of eggs, **(D)** Length of ovariole, **(E)** Number of laid eggs, and **(F)** Hatching rate were analyzed, respectively. The results were shown as mean ± SE. The asterisk ^∗∗^ above the bars represented significant difference (*P* < 0.01).

Knockout of *VgR* resulted in whiter and smaller eggs (444.3 ± 20.4 μm for MUT vs. 536.7 ± 15.8 μm for control) (*t* = 3.579, df = 58, *P* = 0.001) (Figures 6A,C), as well as the shorter ovarioles (7.1 ± 0.5 mm for MUT vs. 10.7 ± 0.5 mm for control) (*t* = 7.407, df = 28, *P* = 0.000) in newly emerged females (Figures 6B,D). The number of eggs laid per female from the MUT strain was not significantly lower than that from the WT strain during the first 3 days of mating (MUT = 138.8 ± 9.1 and WT = 141.5 ± 8.1 eggs) (*t* = 0.222, df = 43, *P* = 0.825) (Figure 6E). However, the hatching rate dropped sharply from 0.9 for WT strain to 0.2 for MUT strain (*t* = 13.461, df = 43, *P* < 0.001) (Figure 6F).

## Discussion

Our study contributed to not only confirming, but also solving, the full coding sequence of *PxVgR* from the previously published genome, which contained some splicing errors (Tang et al., 2014). We also for the first time explored the functions of *VgR* gene on Vg transportation, ovary development, and fecundity in *P. xylostella* to clarify its reproductive functions using CRISPR/Cas9 system.

The *VgR* gene of *P. xylostella* was part of the LDLR superfamily protein with the four typically conserved domains including LBD, EGF, TMD, and CPD, but lacked the OLSD. This result is similar to other insect VgRs, such as *H. armigera* (Zhang et al., 2016), *Spodoptera litura* (Shu et al., 2011), *Solenopsis invicta* (Chen et al., 2004), and *Drosophila melanogaster* (Chen et al., 2010), suggesting that the OLSD motif is not completely conserved in insect VgRs and may be highly species-specific. Four A repeats classes in LBD1 and seven in LBD2 were found in *Px*VgR. In other insect species, the numbers of LDLR repeats in LBD motifs greatly differ from the number found in *P. xylostella* (Cho and Raikhel, 2001; Ciudad et al., 2006; Lin et al., 2015; He et al., 2019). LBDs are a functional domain that mediates the interaction between ligand and receptor, and the repeat regions at the N-terminal of LBDs are used to bind Vg protein molecules (Yamamoto et al., 1984). We speculated that the VgR of insects might have different abilities to bind to Vg due to the different numbers of LDLR repeats in LBDs. The structures of EGF and CPD, which regulate the dissociation and endocytosis processes of VgR, are also species-specific (Tufail and Takeda, 2005, 2007). Lin et al. (2013) reported a lethal effect of EGF mutation of VgR in *B. mori* embryos due to its incapacity to mediate a new Vg transport. Thus, we suggested that the structure and modification characteristics of insect VgRs might be closely related to Vg transport processes and efficiency.

The expression of *PxVgR* mRNA was highest in female adults and lower in eggs and female pupae. This pattern was almost synchronized with the vitellogenesis and egg maturation of *P. xylostella* (Zou, 2015). Similarly, Lin et al. (2013) indicate that the expression dynamics of *VgR* gene in *B. mori* are consistent with its oogenesis, i.e., *VgR* expression level increases from late larval stage at the beginning of vitellogenesis and reaches a peak at pupal stage during oocyte maturation. The transcription of the *VgR* also starts from the previtellogenic stage in *Aedes aegypti* to rise during vitellogenesis and peak after egg maturation (Cho and Raikhel, 2001). But there is also a low expression of *VgR* in more developed oocytes in other insects (Tufail and Takeda, 2005, 2007) and non-insect species (Davail et al., 1998). This phenomenon may be related to the recycling of VgR, that the main synthesis of VgR occurs in the previtellogenic period. *PxVgR* was expressed in the egg stage, while the vitellogenin was no longer ingested, suggesting that VgR was not only a ligand of Vg, but might also participate in other physiological processes, such as embryonic development. Western blot analysis also revealed that the developing expression patterns of *Px*VgR protein were similar to its transcripts detection.

Both qPCR and Western blot analyses indicated that *Px*VgR specifically expressed in the ovaries of *P. xylostella*, which were similar to those in other insects, including *B. mori* (Han et al., 2017), *Nilaparvata lugens* (Lu et al., 2015), and *S. litura* (Shu et al., 2011). Tissue-specific expression profiles of VgRs further confirm their pivotal roles in reproductive regulation (Tufail and Takeda, 2005, 2007; Shu et al., 2011; Lin et al., 2013; Lu et al., 2015). However, some studies have shown that VgRs expression is not limited to the ovary in insects. The VgR is also found to be expressed in head, midgut, and hypopharyngeal glands of *Apis mellifera* (Guidugli-Lazzarini et al., 2008). Roy-Zokan et al. (2015) show that VgR is highly expressed in the head of *Nicrophorus vespilloides*. In addition, the *VgR* expression also significantly increases in fat body of female *Bactrocera dorsalis* 5–6 day post-eclosion, thus ensuring the VgR storage in the fat body (Lin et al., 2015). These findings of extraovarian expressions of insect VgRs may be attributed to the diverse functions in other physiological processes, including labor differentiation, behavior formation, longevity regulation, food storage, and immune response (Corona et al., 2007; Nelson et al., 2007; Guidugli-Lazzarini et al., 2008; Singh et al., 2013).

The CRISPR/Cas9 system is conducive to efficiently and accurately explore the gene functions in insect species, especially in non-model agricultural pests (Huang et al., 2016; Guo et al., 2019), such as Lepidoptera species. This technique has a high potential for developing new plant protection methods through integrated pest management (IPM) in a safe, cost-effective and sustainable manner (Gantz et al., 2015; Xu et al., 2019). Here, we constructed the homozygous mutant strain of *VgR* gene in *P. xylostella* based on the Mendelian law of inheritance and segregation (Liu et al., 2017). We obtained the homozygous mutant with a 5 bp deletion of *PxVgR* gene for the next three generations and established the stable homozygous mutant strain in F4. The lack of *Px*VgR protein was detected using Western blot, immunofluorescence and mass spectrometry for providing more sufficient evidence to ensure the successful knockout.

*PxVg* transcription did not suppress after *PxVgR* knockout, which was similar to other studies showing that silencing of *VgR* did not affect the transcription level of *Vg* (Lu et al., 2015; Upadhyay et al., 2016; Zhang et al., 2016). Although the Vg deposition was obviously reduced, Vg protein was still detected in the eggs laid by the MUT strain *P. xylostella*. This result seems to break the traditional cognition that the specific receptor for Vg is VgR (Tufail and Takeda, 2009; Lin et al., 2013; Han et al., 2017). We speculated that on the one hand, it might be possible that other LDLR family members or genes with similar conserved domains might also take part in Vg transport. Kwankaew et al. (2018) indicate that a unique C-type lectin containing LDLR domain displays the high binding affinity to Vg, which may signify a new function that acts as a receptor for Vg transport in *Fenneropenaeus merguiensis*. On the other hand, it may be due to the synergistic interaction between symbiotic microorganisms and Vg protein (Wei et al., 2017). However, whether Vg can be carried by symbiotic microorganisms for entering into the oocytes remains to be further studied.

The number of eggs laid by *P. xylostella* MUT strain did not significantly decrease when compared to the WT strain, but the hatching rate of MUT sharply dropped. This suggests that the *VgR* gene does not affect the oogenesis, but may participate in the embryonic development of *P. xylostella*. This finding is inconsistent with previous results that *VgR* silencing inhibits the Vg deposition and causes significant reduction in the number of laid eggs in insects, including *Bemisia tabaci*, *N. lugens*, and *H. armigera* (Lu et al., 2015; Upadhyay et al., 2016; Zhang et al., 2016). Other studies, however, have shown that some insects are still able to produce mature eggs with chorion despite lacking vitellogenin (Telfer, 1954; Bownes and Hames, 1977; Yamashita and Irie, 1980). We speculated Vg might not be the major egg yolk precursor protein involved in oogenesis of *P. xylostella*. We also found that *Vg* knockout had no obvious influence on the number of laid eggs of *P. xylostella*, but only on their hatching rate, as well as some works supporting our hypothesis that the types of major yolk precursor protein are species-specific. For example, the transferrin is the major yolk precursor protein in sea urchins, while, in decapod crustaceans, apolipocrustacein is in yolk polypeptides of higher Diptera (Bownes and Pathirana, 2002; Brooks and Wessel, 2002; Avarre et al., 2007).

Knockout of *PxVgR* resulted in the whiter and smaller eggs of *P. xylostella*. Our results were similar to those reported by Lin et al. (2013), where in *B. mori*, *VgR* mutation leads to the white and smaller eggs laying. Previous studies have shown that LDLRs are able to react to multiple types of receptors through ligand recognition and immunoreactivity. For instance, VgR can transport the Vg and other low-density lipoprotein into developing oocytes in chicken (Stifani et al., 1990; Sappington and Raikhel, 1998), as well as recognize various ligands in insect species (Tufail and Takeda, 2009). Therefore, we inferred that the defects of eggs laid by *P. xylostella* with VgR mutation might be not only associated with a decrease in Vg deposition, but other proteins that provided nutrients for egg development. Further studies will be needed to confirm which other proteins also enter into *P. xylostella* eggs via VgR-mediated transport to promote egg development.

## Conclusion

In this work, we successfully knocked out *PxVgR* using the CRISPR/Cas9 system and illuminated its roles in Vg transportation, ovary development, oviposition, and embryonic development of *P. xylostella*. These results suggest that the traditional concepts that VgR is the specific receptor to mediate Vg transport into oocytes and plays a key role in egg formation in most insects may need to be further studied. This is the first time that, using CRISPR/Cas9 technology, the reproductive regulation of *VgR* in *P. xylostella* can be explored. This study lays the foundation for understanding molecular mechanisms of *P. xylostella* reproduction, and for making use of *VgR* as the potential genetic-based molecular target for better control of the *P. xylostella.* Further works need to be carried out to explore the detailed functions of *VgR* on embryonic development and the other factors of Vg transport in *P. xylostella.*

## Data Availability Statement

The datasets generated for this study can be found in the GenBank, *VgR*: MN044389.

## Author Contributions

LP and M-SY designed the study. LP, QW, M-MZ, Y-DQ, and L-NC completed the experiments. Y-LZ, S-JD, and L-LL analyzed the data. LP wrote the first draft of the manuscript. W-YH and GY wrote sections of the manuscript with M-SY and LV. All authors have approved the manuscript and were substantially involved in revision of the manuscript.

## Conflict of Interest

The authors declare that the research was conducted in the absence of any commercial or financial relationships that could be construed as a potential conflict of interest.

## Footnotes

1 http://59.79.254.1/DBM/index.php

2 http://www.ncbi.nlm.nih.gov/gorf/orfig.cgi

3 http://www.cbs.dtu.dk/services/SignalP/

4 http://www.expasy.org

5 http://www.ebi.ac.uk/interpro/

6 http://www.cbs.dtu.dk/services/TMHMM-2.0/

7 http://comp.chem.nottingham.ac.uk/glyco/

8 https://www.atum.bio/eCommerce/cas9/input
