# Supplementary material for: CRISPR/Cas9-Mediated Vitellogenin Receptor Knockout Leads to Functional Deficiency in the Reproductive Development of Plutella xylostella
Source: Front Physiol. 2020 Jan 23;10:1585. doi: 10.3389/fphys.2019.01585 (PMC6989618; doi:10.3389/fphys.2019.01585)
Supplement: Supplementary file 1 [file Data_Sheet_1.PDF]

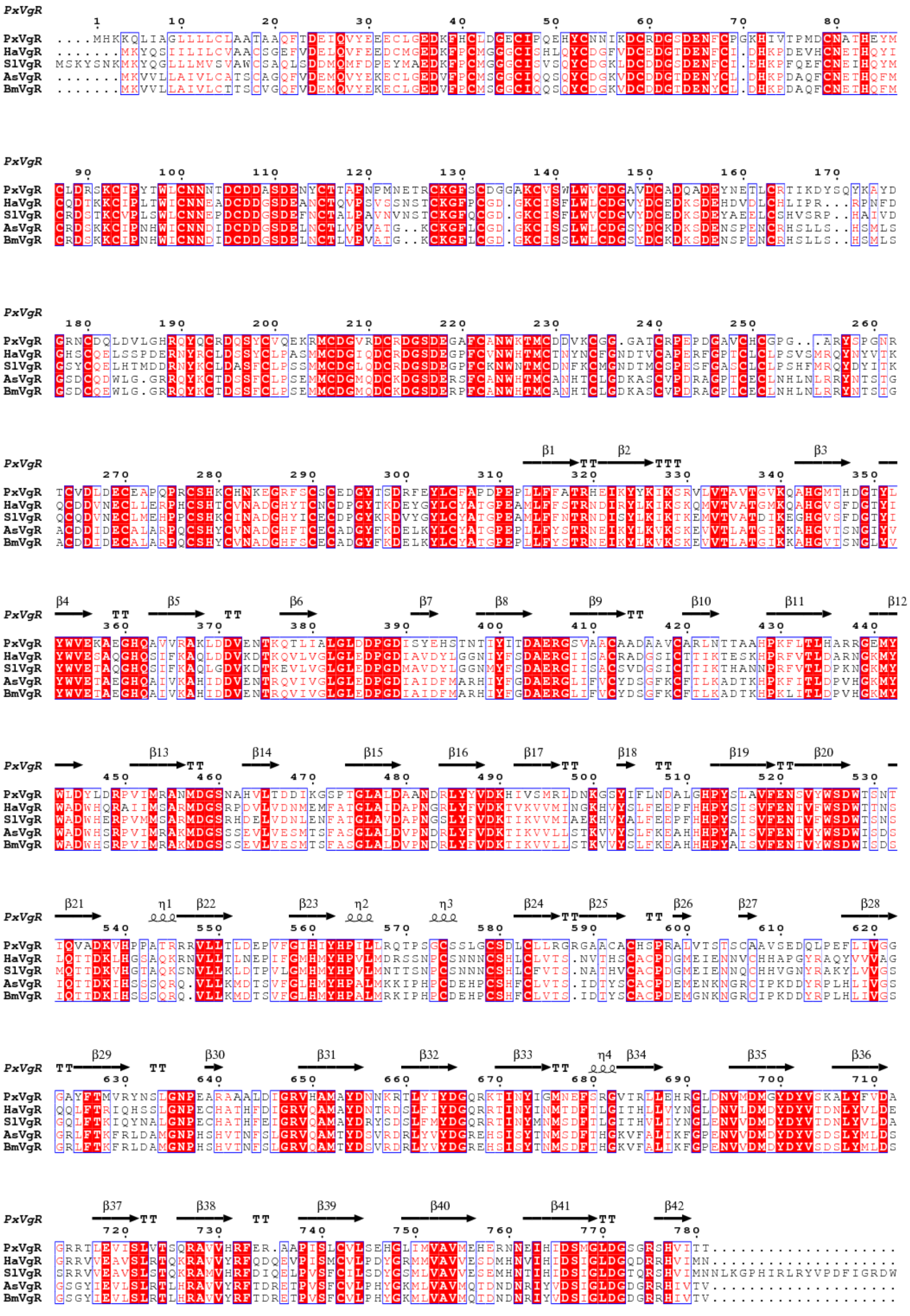

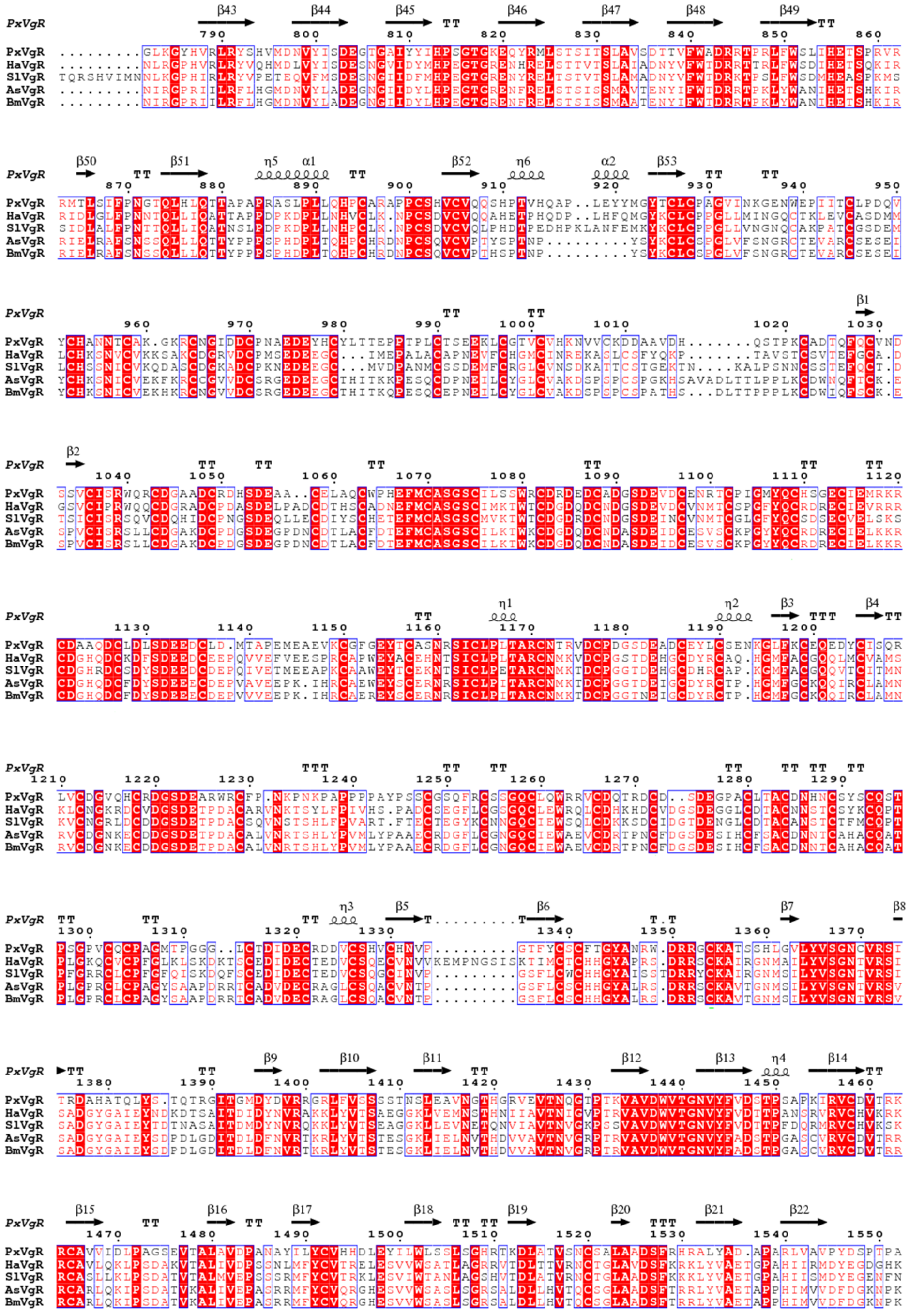

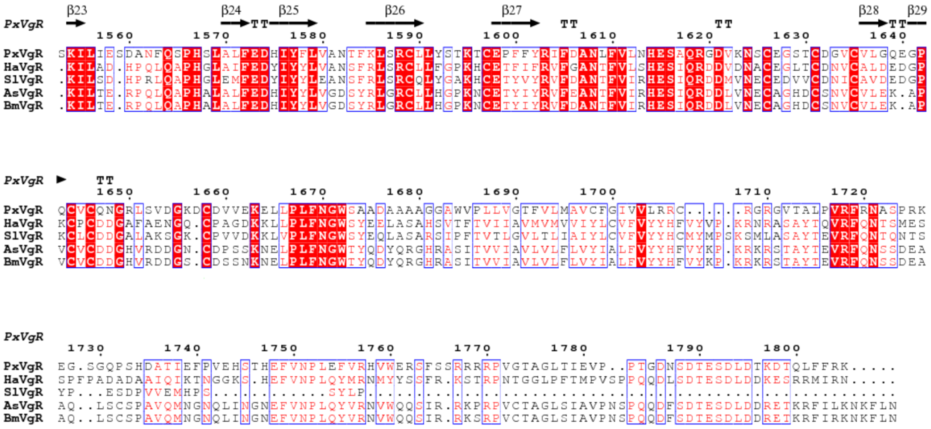

Figure S1 Multiple alignment of amino acid sequences of VgRs in insects.
