# Supplementary material for: CRISPR/Cas9-Mediated Vitellogenin Receptor Knockout Leads to Functional Deficiency in the Reproductive Development of Plutella xylostella
Source: Front Physiol. 2020 Jan 23;10:1585. doi: 10.3389/fphys.2019.01585 (PMC6989618; doi:10.3389/fphys.2019.01585)
Supplement: Supplementary file 2 [file Data_Sheet_2.PDF]

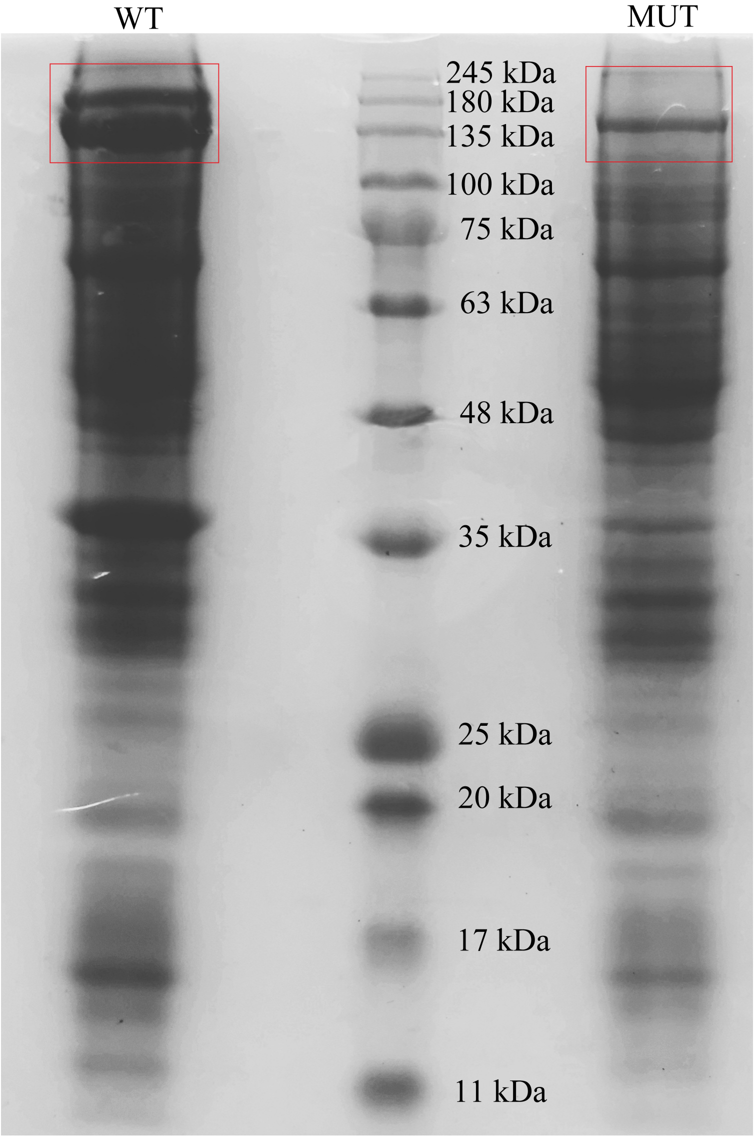

Figure S2 SDS-PAGE of the egg protein of *P. xylostella* from the wild type and VgR mutant strains. Gel section in the red rectangle was excised after stained with Coomassie blue for proteomic identification using HPLC-MS/MS.
