# Supplementary material for: CRISPR/Cas9-Mediated Vitellogenin Receptor Knockout Leads to Functional Deficiency in the Reproductive Development of Plutella xylostella
Source: Front Physiol. 2020 Jan 23;10:1585. doi: 10.3389/fphys.2019.01585 (PMC6989618; doi:10.3389/fphys.2019.01585)
Supplement: Supplementary file 3 [file Data_Sheet_3.PDF]

MSVDDSKRSLLTTHISPAVAATTGRPSHDVTSETTAWPWQDGKL
YEYDVTSHTLATLPEGASAGAALRASLQLRARAGRLLARLLLP
RHARVHQPLPARSLPADLDYKPYTYLDQPFEISVVGGRVVALNV
PATLSAANQNLLKGLISALQVDLTPYRTVRDSHSFFNKETFQGQ
FYKKEIDVTGDCETLYTVAPLAAEWRRELPAFADHDEPVEITKS
KDYGKCHHQVAFNFGIPAGAVWSGVALNPQSTQLIKRHTESRII
AGKQGPIYQAQVTSTVHASPLMYGQQKAEVYSVVKLSLLSIKQ
DDQEEWKLSEKMRAIKNLLYTVSANGVHIDENSSEESTEQIDSI
RRRRASHESDEMLDMWSSQETNDDIANIEHPAYMPMYMVQAK
ENKNAPATVQKLVQEIAQQLQNPNNMPKADTLTKFNIVVRLIA
GMSQEQLALTSRSIEAAKASDIIIKNDMWMVYRDAVAQAGSMP
AFHQIQTWIKTKKLQGEEAAQVVATLPKTLQYPTKDLMIQFFN
MAMCEEVMKQPYLNSTALIAAARFINQGQVNNLTAISYYPTYM
YGRLARRHDGFVVDMILPRLSEDLKQAIERGDSHRAQVVIKAI
GNLGHRAILDVFTPYLQGQVVVSTFLRRLMVDNLYQLASERDH
AVRAVLYSILRNSAEPYEVRLSALQSIFAAQPTVAMMQAMARM
TLDDPSVQVRAALKAIILSAAKLKDHRFRDLAKMADSVKEIVT
KEKFDIVYSQKQMIDFFSQDQDLGFLAVLSSIGSEDSLAPQYLR
YSWWNKVHGWGNKNTIAASMSNVRQFIEYFSEQMNIQKKQE
YKTSDFKAKFSAEKIAEMLQIKRDPEQPLEAAFDIEIMNHQRFL
VFSEADMQQLPREFARGIERLAQGVDFHYTKMLSPARATVMFP
VAMGVPFIYQYKEPTLLHVQGKAKASVTFPGKESKDFATTIDKE
IQITFARNIDGSVGFFDTLSNQYSSAGVVSKIQFNIPIKAQIKIEA
GKLKFNLEPLNPDMDTTVAHFSVWPYTASQAKDSHVPAALDPT
TKVITRSEKVTTVDTKFGLVSTGIQFQAQGYSYSKDFRNFGTM
MKGNDFMSNIIFAAYQKDVALTHYNFKFLGKQSVNKRVSITAV
YGKYYNQKSSVDALKPTDISDVSPDSAARRELLVQRVAAGVTP

ARVQLLDLSASFLGAQPADYVLTASAAQSSVDPKIQYALFAART
HAKLGNSQLNAVGTFMKPEITSMNFPEVLKQEIKTDFNADIKFG
TSGKIQVIGFLERTKKYTEILKNLPRSKQCMSEIAEKNYYQPAC
HQMIKMANSPDFLSASVTYKDVSPSVKNMTYQLYRLARHAGF
WYTEENTLRSTVDGKFDVTLDMSYVQNRFELAVASRAGDLRV
RSGYLPDVALSAAAIYSPMYTSMVDRVVNYYTGHQYMPYCAV
EDNKIKTFSNRSYSYSMGAAWHVVALDDASYAGRRQHDPRQL
VLLARRPVPQHQEIYIFYRTEPGKEMEIEVKPAPEDSNKKGIVKI
TTNLKMISEGELTMYWDEETKMPLLKYYTLADGSMVFSIEQGR
ARVIYDGQRLVVLRKENRGNSRGVCGYMSGDVRDDYLTPAGI
VDAPEHYGASYALREHATPALLELQQQAAKLAYQPAQHYTAIL
AADAEWSQSMQREHVWQHAYRVRYYGASAECRVQQQVQYY
EHHGDTCVSTRQVAACAAQCRGDGYEVQPAEVVCGPRGDKVF
R S Y V E Q I R A G G N P Q V S G V T K I Q Q F R V P K R C L A

Figure S3 The tryptic peptides (red) identified from the egg protein of *P. xylostella* of VgR mutant strain matching to the *P. xylostella* Vg protein.
